# Evaluation of Root Coverage in Gingival Recessions Treated With Collagen Matrix and Platelet-Rich Fibrin: A Systematic Review

**DOI:** 10.7759/cureus.97385

**Published:** 2025-11-20

**Authors:** Pranjali Bhagwat, Richa Kamat, Prajakta Rao, Karishma Ashok, Priyanka Shitole, Sanpreet S Sachdev

**Affiliations:** 1 Periodontics, Bharati Vidyapeeth (Deemed to Be University) Dental College and Hospital, Navi Mumbai, IND; 2 Oral Pathology and Microbiology, Bharati Vidyapeeth (Deemed to Be University) Dental College and Hospital, Navi Mumbai, IND

**Keywords:** collagen membrane, gingival recession, platelet-rich fibrin, root coverage, soft-tissue regeneration

## Abstract

Gingival recession is a common mucogingival defect that results in root exposure, dentinal hypersensitivity, and aesthetic compromise. Various surgical techniques and biomaterials have been employed to achieve predictable root coverage, among which collagen matrices (CM) and platelet-rich fibrin (PRF) have gained clinical prominence. This systematic review aimed to evaluate and compare the efficacy of CM and PRF in achieving root coverage and improving soft-tissue parameters in gingival recession defects. Electronic searches were conducted across PubMed, Web of Science, Scopus, Sciencedirect, EBSCOHost, and Google Scholar databases up to September 2025, following the Preferred Reporting Items for Systematic Reviews and Meta-Analyses (PRISMA) 2020 guidelines. Only randomized controlled trials comparing CM and PRF were included. Six studies involving 210 sites were analyzed qualitatively. Both PRF and CM demonstrated significant improvements in root coverage, gingival thickness, and keratinized tissue width; however, three studies showed superior outcomes with PRF or PRF combined with CM compared to CM alone. Techniques such as the vestibular incision, subperiosteal tunnel access, and pinhole surgical technique, when combined with PRF, provided better soft-tissue outcomes and patient comfort. The overall quality of evidence, evaluated using the Grading of Recommendations Assessment, Development and Evaluation (GRADE) framework, was moderate due to methodological heterogeneity and lack of blinding in outcome assessment. Within the limitations of this review, PRF, either alone or in combination with CM, appears to offer enhanced regenerative outcomes and greater predictability in root coverage procedures compared to CM alone.

## Introduction and background

Gingival recession (GR) is defined as the apical displacement of the marginal gingiva resulting in exposure of the root surface, which may lead to hypersensitivity, root caries, plaque accumulation, and compromised esthetics [1]. While traumatic toothbrushing and periodontal inflammation remain the most frequently cited etiological factors, contemporary clinical trends reveal a growing incidence of recession in patients undergoing orthodontic therapy, particularly clear aligner and labial movement-based treatments that predispose to labial bone dehiscence and soft-tissue thinning [2]. The increasing global use of aligner therapy has therefore renewed clinical attention toward mucogingival stability and the long-term preservation of periodontal phenotype in younger adult populations.

Predictable root coverage (RC) procedures aim to restore the lost soft-tissue margin, reduce hypersensitivity, improve esthetic outcomes, and prevent further apical migration [3]. Over time, several periodontal plastic surgical techniques such as the coronally advanced flap (CAF), tunnel-based approaches, and lateral pedicle grafts have been introduced, with the combination of CAF and a subepithelial connective tissue graft (CTG) remaining the gold standard for Miller’s Class I and II defects due to its high predictability and long-term stability [4]. Nevertheless, CTG is associated with inherent limitations, including donor-site morbidity, limited graft availability, increased surgical time, and heightened postoperative discomfort [5], thereby motivating the exploration of alternative biomaterials that can reduce patient morbidity while maintaining regenerative potential.

Guided tissue regeneration (GTR) techniques using barrier membranes were introduced to address these limitations by preventing epithelial downgrowth and promoting selective repopulation by periodontal ligament cells [6]. Among the biomaterials investigated, collagen matrices (CM) and platelet-rich fibrin (PRF) have gained particular attention as substitutes for autogenous grafts [7]. Xenogeneic CM function as semipermeable scaffolds enabling nutrient diffusion and fibroblast adhesion, while modern bilayer bioresorbable membranes enhance wound stability and angiogenesis with excellent biocompatibility [8,9]. Recent randomized controlled trials (RCTs) further support their use, demonstrating that CM can achieve RC outcomes comparable to CTG with reduced morbidity in minimally invasive techniques such as the modified coronally advanced tunnel (MCAT) procedure [10].

PRF, introduced by Choukroun et al., is a biologically active, second-generation autologous platelet concentrate composed of a fibrin matrix enriched with leukocytes and sustained-release growth factors, such as platelet-derived growth factor (PDGF) and transforming growth factor-β (TGF-β), that promote angiogenesis, fibroblast proliferation, and early tissue remodeling [11]. Its role has expanded across periodontal and peri-implant regeneration, and recent evidence has reported PRF matrices to yield RC outcomes comparable to CTG while improving patient comfort and soft-tissue phenotype [12,13]. Furthermore, advanced PRF formulations have demonstrated enhanced regenerative potential, improved bone preservation, and favorable soft-tissue maturation in periodontal and oral surgical applications [13,14].

Despite the growing body of evidence supporting both CM and PRF, there is no consensus regarding their comparative effectiveness in RC procedures, especially considering the evolution of minimally invasive surgical techniques and the introduction of newer PRF derivatives. Recent clinical trials evaluating CM versus CTG [10] and PRF versus CTG [12] highlight the need to clarify the relative performance of these biomaterials, particularly in the context of patient-centered outcomes, soft-tissue dimensional stability, and regenerative predictability. Therefore, this systematic review aims to comprehensively evaluate and compare the relative efficacy of CM and PRF in achieving RC and soft-tissue enhancement in the management of GR.

## Review

Methodology

Protocol and Reporting Framework

The present systematic review was conducted in accordance with the Preferred Reporting Items for Systematic Reviews and Meta-Analyses (PRISMA) 2020 [15], and the protocol was registered in the PROSPERO database (Reference ID: CRD42025645962). Each step, from literature search to data extraction and quality appraisal, was performed independently by two reviewers (P.B. and R.K.) and verified by a third reviewer (P.R.) for accuracy and consistency.

Focused Research Question

The focused question guiding this review was: “What is the effectiveness of CM compared with PRF in improving RC outcomes in patients presenting with moderate-to-severe GR (≥2 mm)?”

Search Strategy

A comprehensive electronic search was performed across the major scientific literature databases, including PubMed, Web of Science, Scopus, Sciencedirect, EBSCOHost, and Google Scholar, covering literature published up to September 2025. The search strategy combined both Medical Subject Headings (MeSH) terms and free-text keywords, including “collagen,” “platelet-rich fibrin,” and “gingival recession.” The complete PubMed query string and corresponding database terms are illustrated in the Appendices. The search was restricted to articles with full text available in English. To ensure completeness, the reference lists of all included studies and relevant systematic reviews were also screened manually to identify additional eligible trials not retrieved through database searches.

Eligibility Criteria

Eligibility was determined using the PICOS framework (Population, Intervention, Comparator, Outcomes, and Study design). The population comprised adult patients with buccal GR defects in anterior or premolar teeth. The intervention group included studies that used CM (xenogenic or bioresorbable) in root-coverage procedures. The comparator group included studies that used PRF or its derivatives, including advanced (A-PRF), leukocyte-rich (L-PRF), or injectable (I-PRF), either alone or in combination with CM. The primary outcome was percentage RC, and the secondary outcomes were recession height (RH), recession width (RW), gingival thickness (GT), and keratinized tissue width (KTW). Only RCTs published in the English language were included. Case reports, case series, non-randomized trials, animal or in vitro studies, review articles, and conference abstracts were excluded.

Study Selection Process

All titles and abstracts retrieved were screened independently by two reviewers (P.D.B. and R.M.K.) using a two-stage process. In the first stage, duplicates were removed, and irrelevant records were excluded based on title and abstract review. In the second stage, full texts of potentially eligible articles were obtained and evaluated in detail against the predefined inclusion and exclusion criteria. Disagreements between reviewers were resolved through discussion with a third reviewer (P.R.), and consensus was achieved for final inclusion.

Data Extraction and Synthesis

Data extraction was performed using a customized Microsoft Word (Microsoft Corp., Redmond, WA) template specifically designed for this review to ensure uniform data capture. The following parameters were recorded for each study: author and year of publication, country of origin, study design (parallel or split-mouth), sample size, surgical technique used, type of CM and PRF employed, follow-up duration, primary and secondary outcomes assessed, and key quantitative findings. Two reviewers (S.S.S. and K.A.) independently extracted data, and all entries were cross-checked by a third reviewer (P.R.) to ensure accuracy and avoid transcription errors. No attempts were made to contact authors for missing data, as all included studies contained complete information relevant to the outcomes of interest.

Due to heterogeneity in study design, surgical techniques, and biomaterial types, a meta-analysis was not feasible. Instead, a narrative synthesis approach was adopted. The individual findings were grouped and summarized by intervention type (CM alone, PRF alone, or PRF + CM combination) and surgical approach. Comparative outcomes for RC, RH, RW, GT, and KTW were tabulated and described to highlight trends in the direction and magnitude of treatment effects.

Risk of Bias Assessment

The methodological quality of each included RCT was assessed using the Cochrane Risk of Bias 2.0 (RoB 2) tool [16]. The following domains were evaluated: randomization process, deviations from intended interventions, completeness of outcome data, measurement of outcomes, and selection of reported results. Each domain was classified as low risk, some concerns, or high risk, and an overall judgment was assigned based on the highest level of bias identified in each study. All studies were independently assessed by two reviewers (P.S. and K.A.), with consensus reached after discussion.

Certainty of Evidence (GRADE Assessment)

To determine the overall certainty of the body of evidence, the GRADE (Grading of Recommendations, Assessment, Development and Evaluation) framework was applied [17]. As all included studies were RCTs, the initial evidence level for each outcome was set to “high.” The certainty was subsequently downgraded by one level, with methodological limitations, inconsistent effect direction, imprecision due to small sample size, or indirectness of evidence noted.

Results

**Figure 1:**
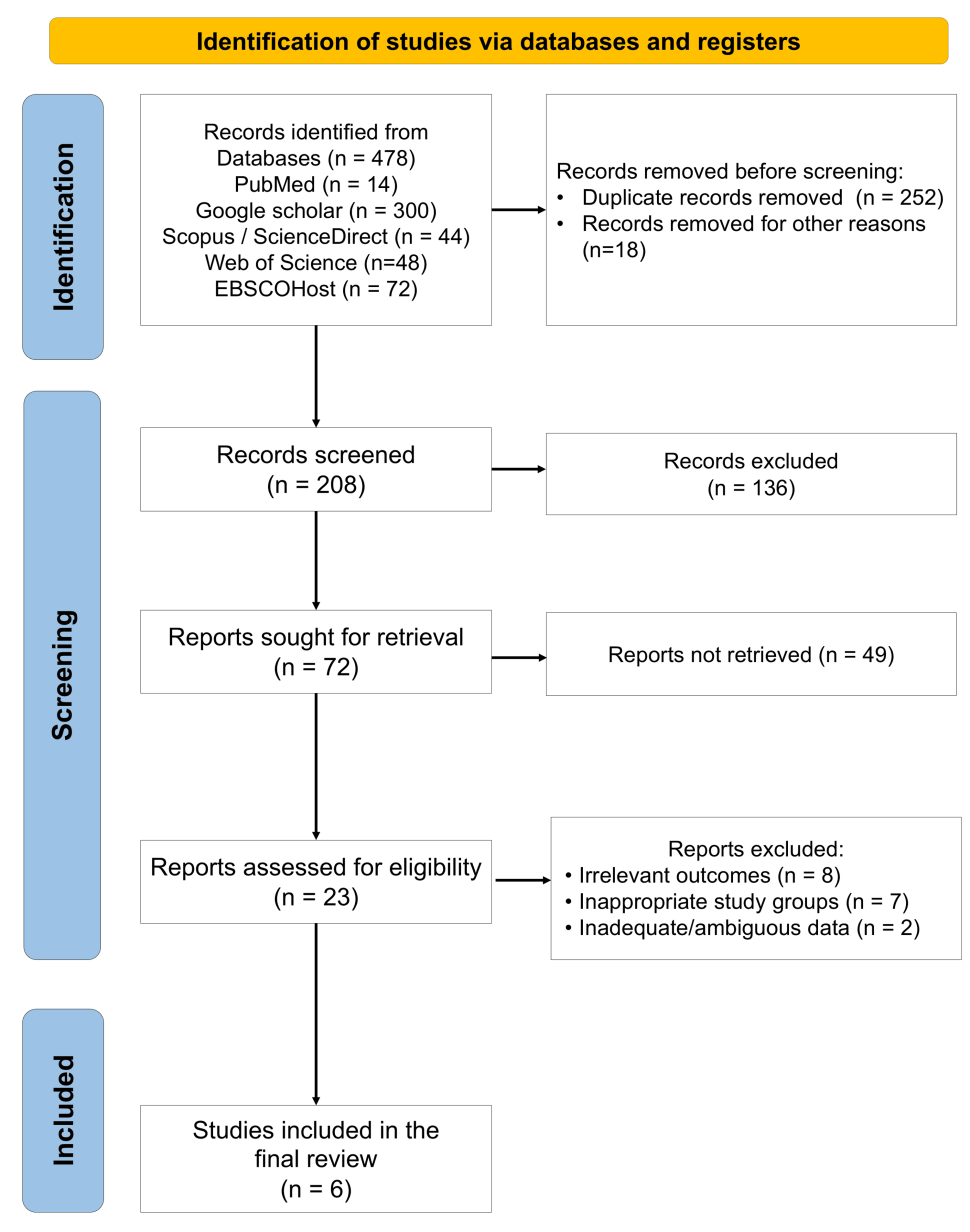
PRISMA flow diagram indicating the selection process of the articles in the present systematic review PRISMA: Preferred Reporting Items for Systematic Reviews and Meta-Analyses

A total of six RCTs conducted between 2020 and 2024 were included in this systematic review (Figure 1) [18-23]. The data extracted from these studies is summarized in Table 1. Collectively, these studies involved 210 GR sites across three countries: India, Yemen, and Egypt. Four studies followed a split-mouth design, whereas two were parallel-group RCTs. The surgical techniques employed varied considerably across studies, including CAF, vestibular incision subperiosteal tunnel access (VISTA), pinhole surgical technique (PST), and open-flap procedures, thereby reflecting heterogeneity in the clinical approach to RC.

**Table 1:** Characteristic data related to methods and outcomes of the studies included in the present systematic review RCT: randomized controlled trial; VISTA: vestibular incision subperiosteal tunnel access; CAF: coronally advanced flap; PRF: platelet-rich fibrin; A-PRF: advanced platelet-rich fibrin; I-PRF: injectable platelet-rich fibrin; L-PRF: leukocyte platelet-rich fibrin; CM: collagen matrix; XCM: xenogenic collagen matrix; PST: pinhole surgical technique; RD: recession depth; RW: recession width; RH: recession height; CAL: clinical attachment level; GT: gingival thickness; KTW: keratinized tissue width; KTT: keratinized tissue thickness; PD: probing depth; PI: plaque index; GI: gingival index; RAL: relative attachment level; RC: root coverage; VAS: visual analogue scale; WKG: width of keratinized gingiva; HKT: height of keratinized tissue; TKT: thickness of keratinized tissue; NRS: numeric rating scale

Author (Year)	Country	Study Design	Intervention	Control	Primary Outcome Measures	Secondary Outcome Measures	Significant Findings	Conclusion
Patra et al. (2022) [18]	India	Split-mouth RCT (n = 40 sites)	VISTA + CM + I-PRF (n = 20)	VISTA + CM (n = 20)	Percentage RC, RD, RW	KTW, KTT, PD, PI, GI, RAL	RC, KTW, and KTT were significantly higher in the I-PRF + CM group (p < 0.05).	The addition of I-PRF to CM enhanced RC, KTW, and KTT compared to CM alone.
Ibrahim et al. (2022) [19]	Egypt	Split-mouth RCT (n = 20 sites)	Open-flap surgery + L-PRF (n = 10)	Open-flap surgery + PRF + CM (n = 10)	Gingival recession (GR), RC	PI, GI, CAL, probing sulcus depth, height of keratinized gingiva	RC and CAL gain were significantly higher in the PRF + CM combination group (p ≤ 0.05).	PRF + CM provided superior RC outcomes compared with PRF alone under open-flap conditions.
Shashikumar et al. (2020) [20]	India	RCT (n = 36 sites)	CAF + L-PRF (n = 18)	CAF + CM (n = 18)	Gingival RH, complete RC	PI, GT, WKG, RAL, RC, VAS	RC and GT values were higher in the L-PRF group, with significantly greater RH reduction (p < 0.05).	CAF + PRF showed slightly improved RC and GT over CAF + CM, though differences were not statistically significant overall.
Raval et al. (2022) [21]	India	RCT (n = 34 sites)	CAF + XCM (n = 17)	CAF + L-PRF (n = 17)	RH, RW	KTW, KTT, Landry’s Healing Index	No statistically significant differences were noted between L-PRF and XCM groups for any variable (p > 0.05).	Both L-PRF and XCM produced comparable improvements in RH, RW, CAL, and healing outcomes.
Durgapal and Shetty (2024) [22]	India	Split-mouth RCT (n = 44 sites)	VISTA + bioresorbable CM (n = 22)	VISTA + A-PRF (n = 22)	Mean RC, RH	GT, WKG, pocket probing depth, GI, CAL	GT was significantly higher in the VISTA + A-PRF group compared to CM (p < 0.05).	Both groups showed significant improvement in RC; however, A-PRF demonstrated greater gain in GT.
Al-Barakani et al. (2024) [23]	Yemen	Split-mouth RCT (n = 36 sites)	PST + A-PRF (n = 18)	PST + resorbable CM (n = 18)	RD, RW	KTW, GT, CAL, PI, NRS	A-PRF group showed significantly greater increase in KTW and GT, and better patient comfort (NRS) (p < 0.05).	Both groups improved in CAL and RD, but PST + A-PRF achieved superior soft-tissue gain and comfort.

The biomaterials used in the included studies represented various forms of PRF and CM. PRF derivatives included A-PRF, L-PRF, and I-PRF, whereas collagen scaffolds included bioresorbable membranes and xenogenic CM. Sample sizes ranged from 20 to 44 sites per study, and follow-up periods ranged from three months to one year, with one study reporting long-term data at five years.

Comparison of RC Outcomes

All six studies demonstrated clinically significant improvements in RC, RH, or RW post-operatively, irrespective of the biomaterial used. Three studies, including Patra et al. (2022), Ibrahim et al. (2022), and Al-Barakani et al. (2024), reported statistically significant superiority of PRF or PRF + CM combinations over CM alone [18,19,23]. In contrast, Raval et al. (2022) and Shashikumar et al. (2020) found no statistically significant difference between the groups, although numerically higher RC percentages were observed with PRF [20,21]. Durgapal and Shetty (2024) reported comparable RC between the A-PRF and CM groups using the VISTA technique; however, a greater increase in GT was observed with A-PRF [22]. Across all studies, PRF, either alone or in combination with CM, tended to yield higher mean percentage RC, greater GT, and greater keratinized tissue gain than CM alone.

Soft-Tissue Dimensional Changes

Soft-tissue parameters such as GT, KTW, and keratinized tissue thickness (KTT) were consistently evaluated as secondary outcomes. Patra et al. (2022) demonstrated significantly greater increases in KTW and KTT with I-PRF + CM compared to CM alone (p < 0.05) [18]. Similarly, Durgapal and Shetty (2024) reported that the A-PRF group achieved a significantly greater gain in GT than the CM group under the VISTA approach, despite both groups showing comparable mean RC [22]. In Al-Barakani et al. (2024), the PST + A-PRF group exhibited a significantly higher increase in KTW and GT values, along with improved NRS comfort scores, when compared to PST + CM [23]. Conversely, Raval et al. (2022) and Shashikumar et al. (2020) reported comparable changes in GT, KTW, and RH between the PRF and CM groups, indicating clinical equivalence but no statistically significant differences [20,21].

Comparative Effect of Combined PRF and CM

Two studies specifically examined the synergistic effect of combining PRF with CM. Patra et al. (2022) used I-PRF in combination with CM and observed significantly higher RC percentages (91.6% vs. 82.3%) and improved tissue thickness parameters compared with CM alone [18]. Similarly, Ibrahim et al. (2022) compared PRF + CM with PRF alone under an open-flap technique, revealing that the combination yielded significantly greater RC and clinical attachment level gain [19]. These findings suggest that the adjunctive use of PRF with CM enhances the regenerative environment at the recession site, thereby improving healing and stability of marginal tissues.

Comparison of CAF and Minimally Invasive Techniques

Among the studies employing CAF, Shashikumar et al. (2020) and Raval et al. (2022) reported favorable outcomes, though not statistically significant, between PRF and CM [20,21]. Both groups demonstrated effective reduction in RH and RW, with L-PRF showing slightly higher mean RC (p > 0.05). In contrast, studies employing minimally invasive approaches such as VISTA or PST tended to yield more favorable clinical responses with PRF, particularly in terms of soft-tissue gain and patient comfort. For example, Durgapal and Shetty (2024) (VISTA) and Al-Barakani et al. (2024) (PST) each reported significant increases in GT and KTW in the PRF groups compared to CM [22,23].

Summary of Significant Findings

Out of six included studies, four demonstrated statistically significant superiority of PRF-based interventions, either alone or in combination with CM, in one or more outcome domains. Specifically, PRF + CM or I-PRF + CM consistently produced a higher percentage of RC, greater GT, and higher KTW than CM alone. The remaining two studies [21,20] reported comparable efficacy between CM and PRF, suggesting that both biomaterials can achieve predictable RC when used with appropriate surgical techniques.

Risk of Bias Assessment

**Figure 2:**
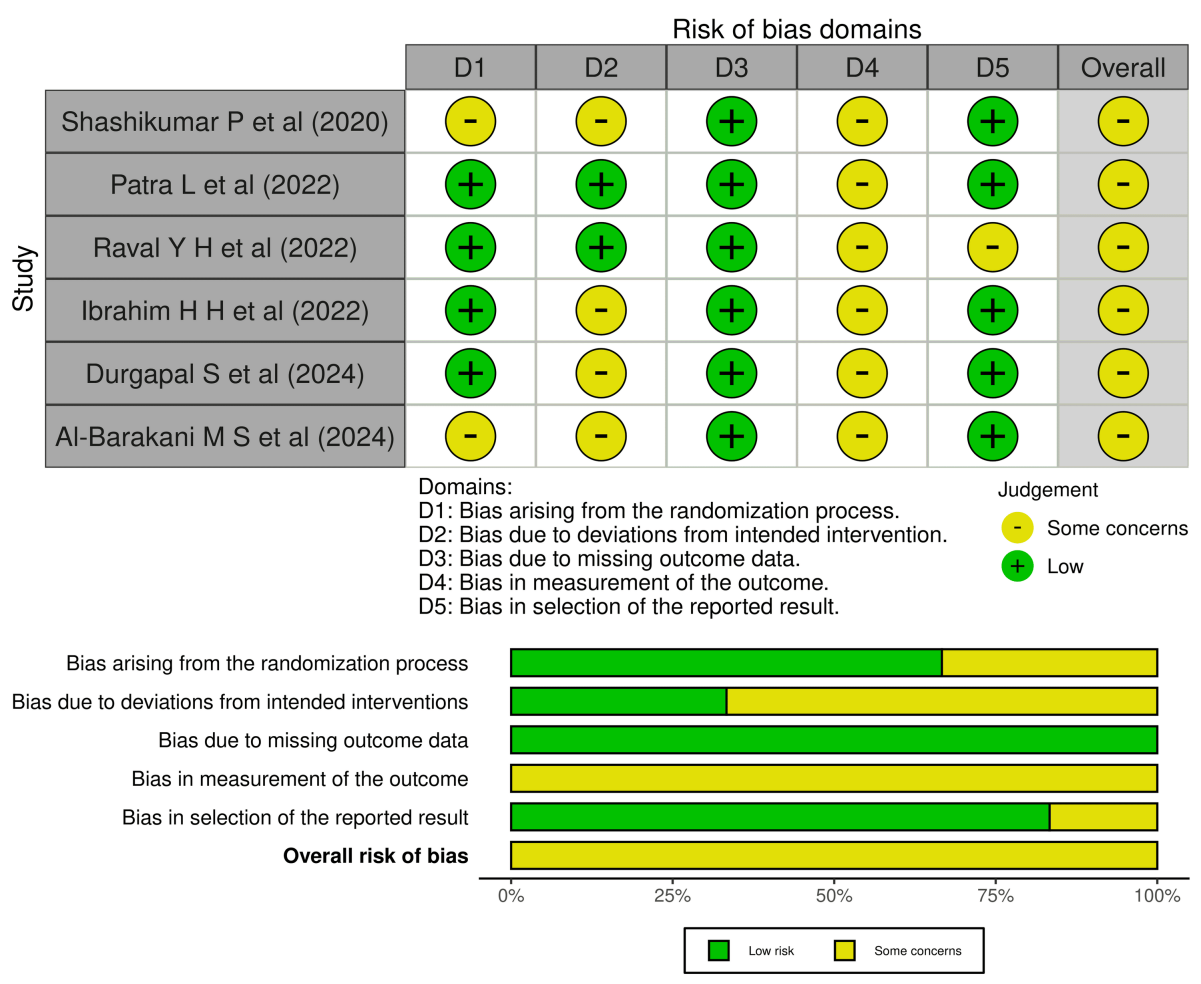
Risk of bias assessment of included randomized controlled trials using the Cochrane RoB 2 tool References [18-23]

The risk of bias analysis using the Cochrane Risk of Bias 2.0 tool indicated that all six included RCTs demonstrated an overall moderate risk of bias (Figure 2). Although most studies implemented appropriate randomization procedures and maintained low attrition, several exhibited methodological weaknesses in other domains. Specifically, the domains of deviations from intended interventions and measurement of outcomes commonly raised concerns, primarily because the majority of studies did not employ blinding of operators or outcome assessors, a critical limitation in surgical trials where subjective evaluation of parameters such as GT, KTW, and RC may introduce detection bias. Overall, the evidence is methodologically sound. Still, it provides moderate overall confidence, reflecting minor yet systematic limitations inherent to clinical periodontal trials evaluating biomaterials such as CM and PRF.

Overall Quality of Evidence

Based on GRADE assessment, the overall certainty of evidence was rated as moderate (Table 2). The downgrade by one level was primarily due to methodological limitations, small sample sizes, and inter-study heterogeneity in surgical design and biomaterial formulation. Despite these limitations, the consistency in direction of outcomes across studies supports the conclusion that PRF, alone or in conjunction with CM, offers a favorable outcome in RC procedures compared with CM alone.

**Table 2:** Summary of quality of evidence assessment ^a^ Due to differences in the surgical techniques used, the effect sizes varied widely; two studies reported 95% confidence intervals (CIs). ^b^ One study demonstrated indirectness in the population, and two studies showed indirectness in the intervention. ^c^ Four studies had no information (NI) regarding CI and optimal information size (OIS). GRADE: Grading of Recommendations Assessment, Development and Evaluation

	Quality assessment				Summary of findings			
Outcome	Risk of bias	Inconsistency	Indirectness	Imprecision	Publication bias	Impact	No. of participants (studies)	Certainty of evidence (GRADE)
Root coverage	Moderate	Moderate^a^	Serious^b^	Serious^c^	Not serious	We are moderately confident in the effect estimate: The true effect is likely to be close to the estimate of the effect, but there is a possibility that it is substantially different	140 (6)	Moderate

Discussion

The present systematic review synthesized available evidence comparing the clinical performance of CM and PRF in RC procedures for GR. Across the six RCTs included, both materials demonstrated satisfactory clinical outcomes in terms of RC, RH reduction, and GT gain. However, the magnitude of improvement varied depending on the type of biomaterial, surgical technique, and follow-up period. Three studies reported significantly superior results with PRF-based interventions, particularly when PRF was used in conjunction with techniques such as pinhole surgery, CAF, and VISTA [20,22,23].

The PST provides a minimally invasive alternative to flap elevation by utilizing existing GT to promote coronal repositioning and healing [24]. In the study by Al-Barakani et al. (2024) [23], PRF integrated with this approach resulted in greater keratinized tissue gain and improved patient comfort compared with CM. Although a case report by Choudhury et al. (2021) [25] found both biomaterials to be effective when used with the same technique, the current findings indicate that PRF’s regenerative potential, driven by autologous growth factors and angiogenic mediators, likely enhances wound healing and stabilization [13,14]. In contrast, CMs function primarily as structural scaffolds that facilitate host cell migration via their collagen-binding sites [9].

The VISTA technique, developed by Zadeh [5], enables access to the recession site via a vestibular incision while preserving the interdental papilla and vascular supply. When combined with PRF, this method, as shown by Durgapal and Shetty (2024) [22], produced greater GT than CM, underscoring the role of PRF as an active biological matrix. However, Al Kababji et al. (2024) [26] observed no significant difference between VISTA alone and VISTA + PRF, while Moraschini and Barboza Edos (2016) [27] similarly reported limited advantage of PRF over conventional flap procedures, potentially due to the rapid degradation of PRF at the surgical site [28].

In CAF-based approaches, one included study demonstrated comparable RC outcomes for PRF and CM [21]. The CAF remains the gold standard for treating Miller’s Class I and II recession defects due to predictable results and enhanced keratinized tissue gain [29]. While CM stabilizes wounds through its porous, fluid-absorptive nature [30], PRF provides a fibrin network that releases TGF-β and PDGF, accelerating tissue remodeling and fibroblast proliferation [31]. Previous reviews have reported inconsistent findings regarding xenogenic CM performance, with Atieh et al. (2016) [32] and Aroca et al. (2009) [14] noting variable RC percentages depending on material composition and surgical technique.

Two studies in this review demonstrated that PRF combined with CM produced superior outcomes compared with either material used alone [18,19]. This synergistic effect may be attributed to PRF's biological activity, which augments CM's mechanical stability, promoting sustained growth factor release and neovascularization. Due to the high leukocyte and cytokine content of I-PRF, the regenerative potential is further enhanced through the “low-speed centrifugation” concept, as described by Mourão et al. (2015) [33].

The present review is limited by the moderate methodological quality of the included trials, heterogeneity in surgical techniques, variability in PRF formulations and CM, and relatively short follow-up periods. Importantly, most studies did not stratify outcomes based on causative factors of GR, such as orthodontic tooth movement, thin periodontal phenotype, traumatic brushing, or periodontal inflammation, which may significantly influence clinical results once incorporated into inclusion criteria, as the reviewer noted. Future research should therefore employ standardized protocols, clearly define etiologic subgroups, utilize uniform biomaterial preparation methods, and incorporate long-term patient-centered outcome measures to better clarify the comparative effectiveness of PRF and CM across different recession scenarios.

Collectively, the reviewed evidence supports PRF, either alone or in combination with CM, as a viable alternative to CTG for RC. However, heterogeneity among studies in terms of PRF type, membrane source, and surgical protocol precluded meta-analysis. According to GRADE evaluation, the certainty of evidence was moderate, mainly due to incomplete blinding, unclear allocation concealment, and methodological variation. None of the studies reported adverse effects, reflecting the biocompatibility of both materials. Future well-designed, standardized RCTs using uniform biomaterial formulations, consistent follow-up intervals, and patient-centered outcome measures are required to establish conclusive evidence on the comparative efficacy of PRF and CM in GR management.

## Conclusions

Within the limitations of this systematic review, both CM and PRF were found to be effective in achieving RC and improving soft-tissue parameters in the management of GR. However, PRF, either alone or in combination with CM, demonstrated superior outcomes in GT, keratinized tissue gain, and percentage RC compared with CM alone. The quality of evidence was moderate, primarily due to methodological variability and limited sample sizes among included studies. Future RCTs with standardized protocols and longer follow-up durations are warranted to validate these findings and establish definitive clinical recommendations.

## Appendices

PubMed query string and corresponding database terms

Search: ((collagen) AND (platelet-rich fibrin)) AND (gingival recession) ("collagen"[Supplementary Concept] OR "collagen"[All Fields] OR "collagen"[MeSH Terms] OR "collagens"[All Fields] OR "collagen s"[All Fields] OR "collagenation"[All Fields] OR "collagene"[All Fields] OR "collageneous"[All Fields] OR "collagenic"[All Fields] OR "collagenization"[All Fields] OR "collagenized"[All Fields] OR "collagenous"[All Fields]) AND ("platelet rich fibrin"[MeSH Terms] OR ("platelet rich"[All Fields] AND "fibrin"[All Fields]) OR "platelet rich fibrin"[All Fields] OR ("platelet"[All Fields] AND "rich"[All Fields] AND "fibrin"[All Fields]) OR "platelet rich fibrin"[All Fields]) AND ("gingival recession"[MeSH Terms] OR ("gingival"[All Fields] AND "recession"[All Fields]) OR "gingival recession"[All Fields]) Translations collagen: "collagen"[Supplementary Concept] OR "collagen"[All Fields] OR "collagen"[MeSH Terms] OR "collagens"[All Fields] OR "collagen's"[All Fields] OR "collagenation"[All Fields] OR "collagene"[All Fields] OR "collageneous"[All Fields] OR "collagenic"[All Fields] OR "collagenization"[All Fields] OR "collagenized"[All Fields] OR "collagenous"[All Fields] platelet-rich fibrin: "platelet-rich fibrin"[MeSH Terms] OR ("platelet-rich"[All Fields] AND "fibrin"[All Fields]) OR "platelet-rich fibrin"[All Fields] OR ("platelet"[All Fields] AND "rich"[All Fields] AND "fibrin"[All Fields]) OR "platelet rich fibrin"[All Fields] gingival recession: "gingival recession"[MeSH Terms] OR ("gingival"[All Fields] AND "recession"[All Fields]) OR "gingival recession"[All Fields]
